# Missed opportunities to prevent cardiovascular disease in women with prior preeclampsia

**DOI:** 10.1186/s12905-020-01074-7

**Published:** 2020-10-01

**Authors:** Alina Brener, Irene Lewnard, Jennifer Mackinnon, Cresta Jones, Nicole Lohr, Sreenivas Konda, Jennifer McIntosh, Jacquelyn Kulinski

**Affiliations:** 1 Department of Internal Medicine, Division of Cardiology, University of Illinois at Chicago, Chicago, IL USA; 2 Department of Obstetrics and Gynecology, Lowell General Hospital, Lowell, MA USA; 3 Department of Internal Medicine, Medical College of Wisconsin, Milwaukee, WI USA; 4 Department of Obstetrics, Gynecology and Women’s Health, Division of Maternal-Fetal Medicine, University of Minnesota Medical School, Minneapolis, MN USA; 5 Department of Internal Medicine, Division of Cardiology, Medical College of Wisconsin, Milwaukee, WI 53226 USA; 6 Division of Epidemiology and Biostatistics, University of Illinois at Chicago, Chicago, IL USA; 7 Department of Obstetrics, Gynecology and Women’s Health, Division of Maternal Fetal Medicine, Milwaukee, WI USA

**Keywords:** Cardiovascular disease, Preeclampsia, Hypertensive disease of pregnancy, Primary prevention

## Abstract

**Background:**

Cardiovascular disease (CVD) is the leading cause of death in women in every major developed country and in most emerging nations. Complications of pregnancy, including preeclampsia, indicate a subsequent increase in cardiovascular risk. There may be a primary care provider knowledge gap regarding preeclampsia as a risk factor for CVD. The objective of our study is to determine how often internists at an academic institution inquire about a history of preeclampsia, as compared to a history of smoking, hypertension and diabetes, when assessing CVD risk factors at well-woman visits. Additional aims were (1) to educate internal medicine primary care providers on the significance of preeclampsia as a risk factor for CVD disease and (2) to assess the impact of education interventions on obstetric history documentation and screening for CVD in women with prior preeclampsia.

**Methods:**

A retrospective chart review was performed to identify women ages 18–48 with at least one prior obstetric delivery. We evaluated the frequency of documentation of preeclampsia compared to traditional risk factors for CVD (smoking, diabetes, and chronic hypertension) by reviewing the well-woman visit notes, past medical history, obstetric history, and the problem list in the electronic medical record. For intervention, educational teaching sessions (presentation with Q&A session) and education slide presentations were given to internal medicine physicians at clinic sites. Changes in documentation were evaluated post-intervention.

**Results:**

When assessment of relevant pregnancy history was obtained, 23.6% of women were asked about a history preeclampsia while 98.9% were asked about diabetes or smoking and 100% were asked about chronic hypertension (*p* < 0.001). Education interventions did not significantly change rates of screening documentation (*p* = 0.36).

**Conclusion:**

Our study adds to the growing body of literature that women with a history of preeclampsia might not be identified as having increased CVD risk in the outpatient primary care setting. Novel educational programming may be required to increase provider documentation of preeclampsia history in screening.

## Background

Cardiovascular disease (CVD) is the leading cause of death in women in every major developed country and in most developing nations [1]. There are unique CVD risk factors for women, many related to pregnancy and/or other hormonal influences. Complications of pregnancy, including preeclampsia, indicate a subsequent increase in cardiovascular risk [2–5]. Preeclampsia at term is an equivalent risk factor for future CVD, but significantly exceeds traditional risk factors when its onset is pre-term or when it recurs in multiple pregnancies, conferring an eight to nine-fold increased risk of CVD [6, 7].

Since 2011, the American Heart Association (AHA) has recognized preeclampsia as a “failed stress test” for heart disease [8]. The 2011 AHA guidelines list preeclampsia, gestational hypertension, and gestational diabetes as risk factors for CVD [8]. The AHA recommends women with a history of these pregnancy complications be referred to a primary care provider or cardiologist by their obstetric providers [8]. Additionally, the guidelines recommend all healthcare providers ask about adverse pregnancy outcomes as part of the medical interview. Similarly, the 2013 American College of Obstetricians and Gynecologists (ACOG) Hypertension in Pregnancy Task Force guidelines recommend early screening with lab testing and lifestyle modification in women who are at particularly high risk of CVD based on a history of preeclampsia requiring delivery prior to 37 weeks and/or recurrent preeclampsia [9].

Prior studies show a primary care provider knowledge gap regarding preeclampsia as a risk factor for CVD ([10, 11]. With publication of the AHA and ACOG guidelines in 2011 and 2013, respectively, the primary aim of our study was to determine how often internal medical physicians inquire about a history of preeclampsia, compared to inquiry on smoking, hypertension and diabetes, when assessing CVD risk factors at well-woman visits. Our hypothesis was that a history of preeclampsia would be elicited significantly less frequently than a history of traditional risk factors for CVD, reflecting the provider knowledge gap established in the literature. Additional aims of our study were (1) to educate internal medicine primary care providers on the significance of preeclampsia as a risk factor for CVD disease and (2) to assess the impact of education interventions on obstetric history documentation and on screening for CVD in women with prior preeclampsia.

## Methods

Institutional review board approval was obtained prior to the initiation of all components of this study. A retrospective chart review was performed to identify women ages 18–48 with at least one prior obstetric delivery. Women who were seen by internal medicine providers at Medical College of Wisconsin (MCW) affiliated clinics for a well-woman visit between January 1, 2013 and May 31, 2016 were included (*n* = 89). Only a single visit per subject was included. Clinical records for this study were obtained by using the Cohort Discovery Tool (CDT). CDT is an informatics framework that pulls clinical research data warehouse, which contains the data sets of all medical record information pulled from patients treated within the Froedtert Hospital, Medical College of Wisconsin, and affiliated provider networks. The tool allows researchers to perform preliminary identification of subjects by searching the specific diagnostic criteria to identify patient records that meet inclusion criteria of the study. The data sources accessed through the CDT link data from electronic medical records and physician billing systems. A manual electronic medical record review was then performed by the study team for the data points included in the analysis. Clinical information collected included patient demographics (age, race, gravidity and parity, time since pregnancy, BMI), major medical conditions (past medical history of diabetes, chronic hypertension, obesity, hyperlipidemia, tobacco use), and pregnancy complications (preterm and term preeclampsia, fetal growth restriction, preterm delivery, and placental abruption). We specifically evaluated how frequently a history of preeclampsia was documented versus a history of traditional risk factors for CVD (smoking, diabetes, and chronic hypertension) by reviewing the well-woman visit notes, past medical history, obstetric history, and the problem list in the electronic medical record.

Educational teaching sessions (presentation with Q&A session) were given to internal medicine physicians at clinic sites. At clinics where in-person teaching sessions were not possible, the educational slide presentations (Additional file 2) were sent to physicians via email (Additional file 3) with a read-receipt link. The read receipt served as confirmation that providers received the material, but was not used to track individual provider participation, nor could it be used to confirm review of the slides.

A second retrospective chart review was subsequently performed using data obtained during the post-intervention period from 12/5/16 to 4/19/17. The electronic medical records of 164 women seen at the same clinics were reviewed to assess the efficacy of the interventions. Clinical records were again obtained from the electronic medical record using the same study inclusion/exclusion criteria. The same data collection tool was used for this review.

For data collected pre-intervention, statistical analysis was performed using a binomial proportions test to compare rates of documented history of preeclampsia versus documentation of traditional CVD risk factors (smoking, diabetes, or chronic hypertension). A two-sample T-test was used to test the difference between post and pre-interventions means of numerical variables (age, BMI, gravidity, parity). Two sample T-interval was used to derive the confidence interval for the difference between post and pre-interventions means. In the study, the patients were independent individual units with different patients in pre-intervention and post-intervention group. Two sample Z-test or Fisher Exact Test was used to test the difference between post and pre-interventions proportions of binary variables (Caucasian, African American, Hispanic, Asian). Two sample Z-interval was used to derive the confidence interval for the difference between post and pre-interventions proportions. Assuming a two-sided alpha = 0.05 and *n* = 89, the exact Binomial test for proportions for independent samples has at least 80% power to reject the null hypothesis that the same proportion of patients were asked about diabetes and PreE in pre-intervention group. Power calculation was chosen for diabetes because it is a risk factor that has a significant  impact on cardiovascular health. The same calculations produced much higher power 99% in post intervention group with *n* = 164. Statistical significance was defined as *p*-value < 0.05.

## Results

**Fig. 1:**
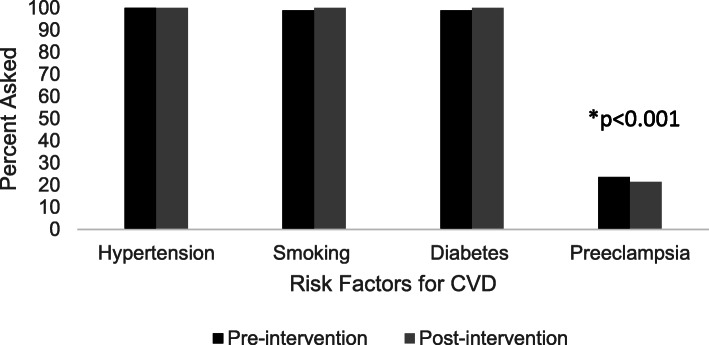
Percent ascertainment of CVD risk factors during well-women visits pre- and post-intervention. *Compared to each traditional CVD risk factor, including hypertension, smoking, diabetes

**Fig. 2:**
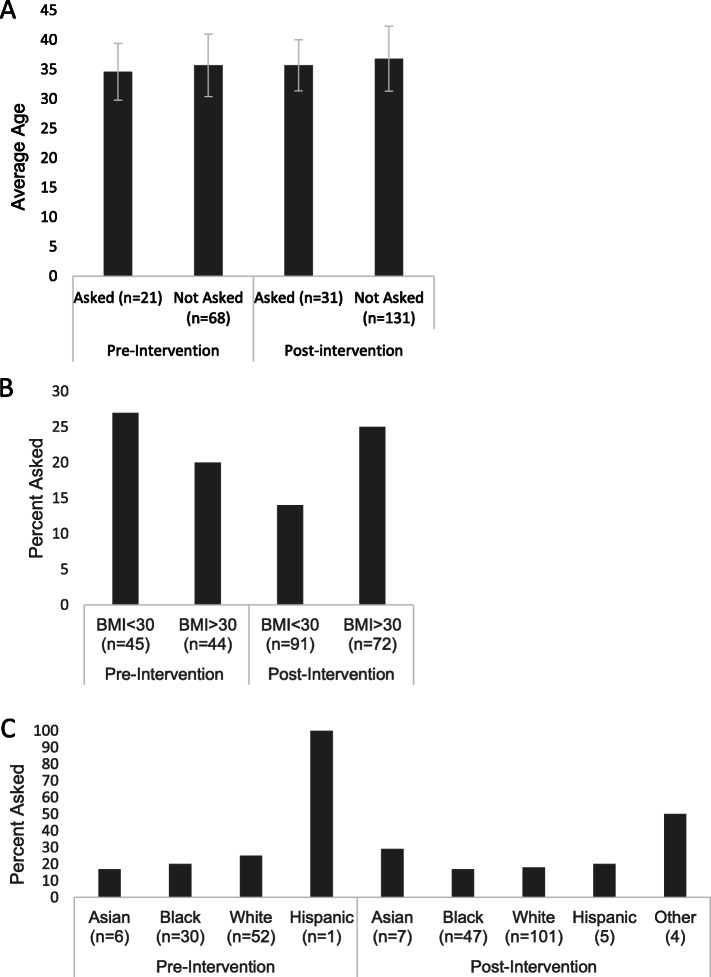
Characteristics of subjects asked (or not) about history of preeclampsia based on: **a**: Age. **b**: BMI. **c**: Race. There was no statistical significance seen in the results above

**Fig. 3:**
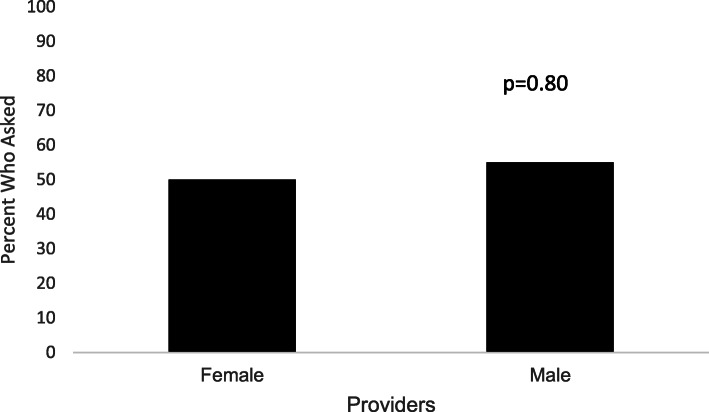
Percent of providers, by gender, who ascertained a history of preeclampsia (*n* = 89)

Demographic data are displayed in Table 1. The (pre-intervention) cohort mean age was 35.4 +/− 5.2 years with 58% Caucasians and 34% African Americans. Mean BMI was 31.5 +/− 10.3 with an average gravity and parity of 3 +/− 1 and 2 +/− 1, respectively. Pre- and post-intervention groups had the same statistical demographics which demonstrated no selection bias. The post-intervention cohort was similar. When assessment of pregnancy history was obtained, 23.6% of women were asked about a history of preeclampsia while 98.9% were asked about diabetes or smoking and 100% were asked about chronic hypertension (Fig. 1). This was significant across each individual comparison group (all *p* < 0.001). There was no significant difference in the mean age, BMI, or race of patients who were asked about preeclampsia pre- and post-intervention compared to those who were not asked (all *p* = NS, Fig. 2). Of note, a sub-analysis (Fig. 3) identified no gender difference among providers who inquired about a history of preeclampsia during the visit (50% of female providers and 55% of male providers, *p* = 0.80).

**Table 1:** Subject characteristics pre- and post-intervention

	Pre-Intervention (*n* = 89)	Post-Intervention (*n* = 164)	Difference (95% CI)	***p***-value
Age	35.2 ± 5.2	36.5 ± 5.5	1.09(− 0.31,2.49)	0.13
BMI	31.5 ± 10.3	31.6 ± 10.2	0.01(− 2.66,2.66)	0.99
Caucasian	52 (58%)	101 (62%)	0.04(−0.10,0.16)	0.24
Hispanic	1 (1%)	5 (3%)	0.02 (0.02,0.05)	0.67
African American	30 (34%)	47 (29%)	−0.05(−0.17,0.07)	0.41
Asian	6 (7%)	7 (4%)	0.03(−0.04,0.08)	0.39
Average Gravidity	2.9 ± 1.5	3.0 ± 1.7	0.10(−0.30,0.53)	0.60
Average Parity	2.2 ± 1.1	2.2 ± 1.2	0.01(−0.29,0.30)	0.97

There were no significant differences between the pre-intervention and post-intervention cohort

When examining the education intervention, it is notable that only 69% of providers who received the presentation by email opened and potentially reviewed the information. There was no significant difference in preeclampsia history screening before compared to after the in-person educational intervention at any clinic site (Supplemental Figure 1). When comparing all clinics pre-intervention and post-intervention (in-person and email), there was no significant difference (all *p*-values > 0.05).

## Discussion

Our study demonstrates that internal medicine providers are not adequately documenting obstetric history during well-woman visits despite ACC/AHA and ACOG guidelines. The 2018 AHA/ACOG Presidential Advisory provides recommendations for identifying women at elevated cardiovascular risk through collaboration with obstetricians and gynecologists [5]. Pregnancy-specific CVD risk factors now include gestational hypertension, preeclampsia, eclampsia, gestational diabetes mellitus, preterm delivery, and low birth weight for gestational age. No official recommendations from other clinical organizations more often referenced by internal medicine providers, such as the U.S. Preventative Services Task Force (USPSTF), exist at this time.

In primary care settings, providers depend heavily on coronary heart disease (CHD) risk calculators such as the Framingham risk score (FRS), which may falsely stratify many young women into a low- risk group. Michos, et al. showed that a majority of women (non-diabetics, mean age 55) with significant coronary artery calcium on imaging were incorrectly classified as low-risk using FRS alone [12]. Importantly, the FRS calculator does not include obstetric history in the calculation. Since preeclampsia is an established risk factor for CVD, the FRS calculator does not effectively risk-stratify women with adverse pregnancy outcomes for preventive interventions. We caution primary care providers that strict reliance on FRS may not identify all women at risk for CHD in the primary prevention setting.

Although no guidelines specify the optimal tool for risk-stratifying patients with adverse pregnancy outcomes, a detailed obstetric history is necessary to determine if any additional CVD risk exists. Work- up considerations with increased risk may include assessing family history, evaluating metabolic markers such as high-sensitivity CRP, and utilizing quantitative tools such as coronary artery calcium scoring or the ankle-brachial index [13]. Studies suggest that women with a history of preeclampsia have increased odds of subclinical atherosclerosis on imaging, characterized by coronary artery calcium (CAC) score, compared to women with normotensive pregnancies at 45–55 years of age [14–16]. In order to improve CVD screening and primary prevention for women, healthcare systems should have an established, cost-effective referral process for patients with adverse pregnancy outcomes. Obstetric providers should inform patients with a preeclampsia diagnosis of increased CVD risk and the need for follow-up with a primary care physician or cardiologist. Unfortunately, studies have shown that many women with a history of preeclampsia are not properly educated about their future health risks, possibly due to lack of provider education and/or awareness of these risk factors [10, 11]. Studies have shown provider awareness of preeclampsia as a risk factor for CVD ranged between 23 and 56% in a US study of internists and obstetricians and 54% in a Canadian study for maternity care providers [10, 11].

Lifestyle interventions guided by American Heart Association Life’s Simple 7 should be emphasized by providers as a primary prevention strategy, whenever possible, for women with adverse pregnancy outcomes [17]. Ideally, this should include an interdisciplinary approach to address weight management, an important part of CVD risk management. Studies show that postpartum weight retention and changes impact cardiometabolic risk in diverse populations [18, 19]. Common themes that emerged from prior studies were a need for cost-effective longitudinal intervention, identifying barriers to healthcare attendance, and developing proactive strategies to promote participation [20, 21]. Since lifestyle interventions are time-consuming and challenging to balance with the needs of a growing family, both telehealth and in-person visits should be considered [20, 22]. Challenges with postpartum weight loss, preeclampsia, and heart disease are more prevalent in the racial/ethnic minorities than their Caucasian counterparts [23–26], and extra efforts should be made to assist these high-risk mothers with lifestyle interventions.

Recommendations for blood pressure optimization should also be addressed in this population. Current guidelines recommend treating Stage 1 hypertension (systolic blood pressure of 130–139 mmHg or a diastolic blood pressure of 80–89 mmHg) based on clinical atherosclerotic cardiovascular disease (ASCVD) or estimated 10-year ASCVD risk of ≥ 10% using the ACC/AHA Pooled Cohort Equation [23]. The Pooled Cohort Equation, however, do not include adverse pregnancy outcomes and may therefore underestimate risk for some women [23]. Progress was made with the release of the 2019 ACC/AHA Guideline on the Primary Prevention of Cardiovascular Disease, which now incorporates the use of risk-enhancing factors, such as pregnancy-associated conditions, to guide decisions about preventive interventions, such as statin therapy. Lifestyle guidance should be given to all patients with elevated blood pressures, and additional, modifiable risk factors for hypertension should be assessed, including obesity, excess sodium intake, low-fiber diet, physical inactivity, excessive alcohol intake, and sleep apnea [5].

Primary prevention is not complete without addressing hyperlipidemia. The 2018 ACC/AHA Guideline on the Management of Blood Cholesterol recognize preeclampsia as a risk-enhancing factor [24]. Moderate statin therapy is now recommended in 40–75-year-old, non-diabetic women with a history of preeclampsia and a 10-year ASCVD risk of 7.5–19.9%. If ASCVD risk is 5–7.5%, statin therapy can be initiated after a risk/benefit discussion. Although statin therapy is currently contraindicated in pregnancy, this contraindication was based on manufacturers’ lack of investigation for use during pregnancy. Multiple studies have demonstrated that the risk of congenital malformations with pravastatin is negligible and likely safe to use in pregnancy [25–29]. A pilot study performed by Constantine et al. preliminarily demonstrated safety and benefit in using pravastatin for preeclampsia prevention in high-risk pregnant women [30]. A large clinical trial is currently underway (clinical trial NCT01717586).

Finally, the utility of aspirin for CVD prevention in patients with a history of preeclampsia is unknown. ACOG and USPSTF recommend use of low-dose aspirin during pregnancy after 12 weeks of gestation for the prevention of preeclampsia in high-risk women [31]. However, there is no study to date which looks at continuing aspirin postpartum for CVD prevention in young women with history of preeclampsia. Per 2019 ACC/AHA recommendations, low-dose aspirin for primary prevention of CVD gets a weak IIb recommendation and might be considered among select adults 40–70 years of age who are at higher ASCVD risk and without increased bleeding risk [13].

Our educational interventions were not successful in improving documentation of preeclampsia screening at well-woman visits. Previous studies looking at cardiovascular guideline implementation in the primary care setting noted that provider education does have small to moderate benefit in physician adherence to practice guidelines [32]. Provider education may require a variety of interventions to be effective including education workshops, meetings, lectures, education outreach visits and distribution of educational material. Prior studies also had 6–12-month follow-up [32], and one of the reasons for lack of statistical significance in our study could be the short follow-up time. Bundling education interventions, for example, between lectures and workshops could have proven more effective. While we utilized read-receipt to ensure the providers open an email with the education slides, the use of conventional email messaging with a slide-set attachment could have also decreased provider engagement and subsequent implementation of the intervention [33].

One limitation of our study includes the lack of generalizability outside a large academic primary care system. Additional research should examine screening rates in private practice primary care programs, as well as in rural communities. In addition, it is difficult to ascertain if lack of documented screening truly indicates lack of screening, or limited ability in the clinical visit template to identify performed screening.

## Conclusion

Our study adds to the growing body of literature that women with a history of preeclampsia are not consistently being identified as having increased CVD risk in the outpatient primary care setting. As cardiovascular disease remains a leading cause of death in women, there needs to be more coordinated effort between specialties to improve identification of women at risk and to implement effective primary-prevention strategies. Future studies should investigate if the use of smart phrases (also known as dot phrases) and best practice alerts could help identify women with adverse pregnancy outcomes and should also examine novel educational approaches to increase screening knowledge.

## Supplementary information

**Additional file 1: Figure S1.** Percent ascertainment of preeclampsia history during well-women visits by clinic, pre- and post-intervention. All *p* > 0.05.

**Additional file 2.** Educational PowerPoint slides for internal medicine providers

**Additional file 3.** Copy of email sent to internal medicine providers with PowerPoint slides attached.

## Data Availability

The datasets used and/or analyzed during the current study are available from the corresponding author on reasonable request.

## Abbreviations

CVD: Cardiovascular disease
AHA: American Heart Association
ACC: American College of Cardiology
ACOG: American College of Obstetricians and Gynecologists
CDT: Cohort Discovery Tool
MCW: Medical College of Wisconsin
USPSTF: U.S. Preventative Services Task Force
CHD: Coronary heart disease
FRS: Framingham risk score
CAC: Coronary artery calcium
ASCVD: Atherosclerotic cardiovascular disease
