# Supplementary material for: Missed opportunities to prevent cardiovascular disease in women with prior preeclampsia
Source: BMC Womens Health. 2020 Oct 1;20:217. doi: 10.1186/s12905-020-01074-7 (PMC7528479; doi:10.1186/s12905-020-01074-7)
Supplement: Supplementary file 2 — Additional file 2. Educational PowerPoint slides for internal medicine providers [file 12905_2020_1074_MOESM2_ESM.pptx]

## Slide 1
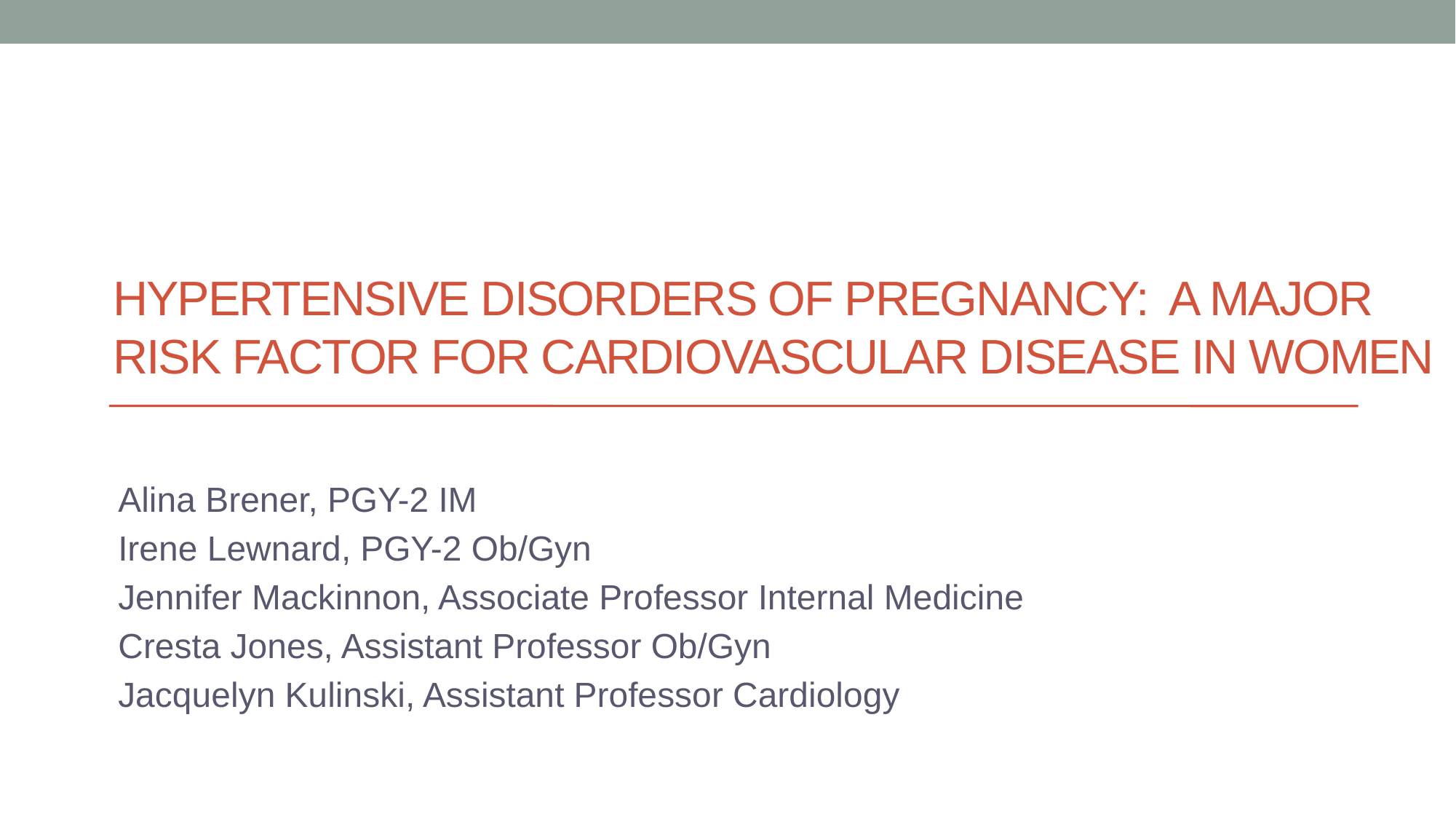

# Hypertensive Disorders of Pregnancy: A Major Risk Factor for Cardiovascular Disease in Women
Alina Brener, PGY-2 IM
Irene Lewnard, PGY-2 Ob/Gyn
Jennifer Mackinnon, Associate Professor Internal Medicine
Cresta Jones, Assistant Professor Ob/Gyn
Jacquelyn Kulinski, Assistant Professor Cardiology

## Slide 2
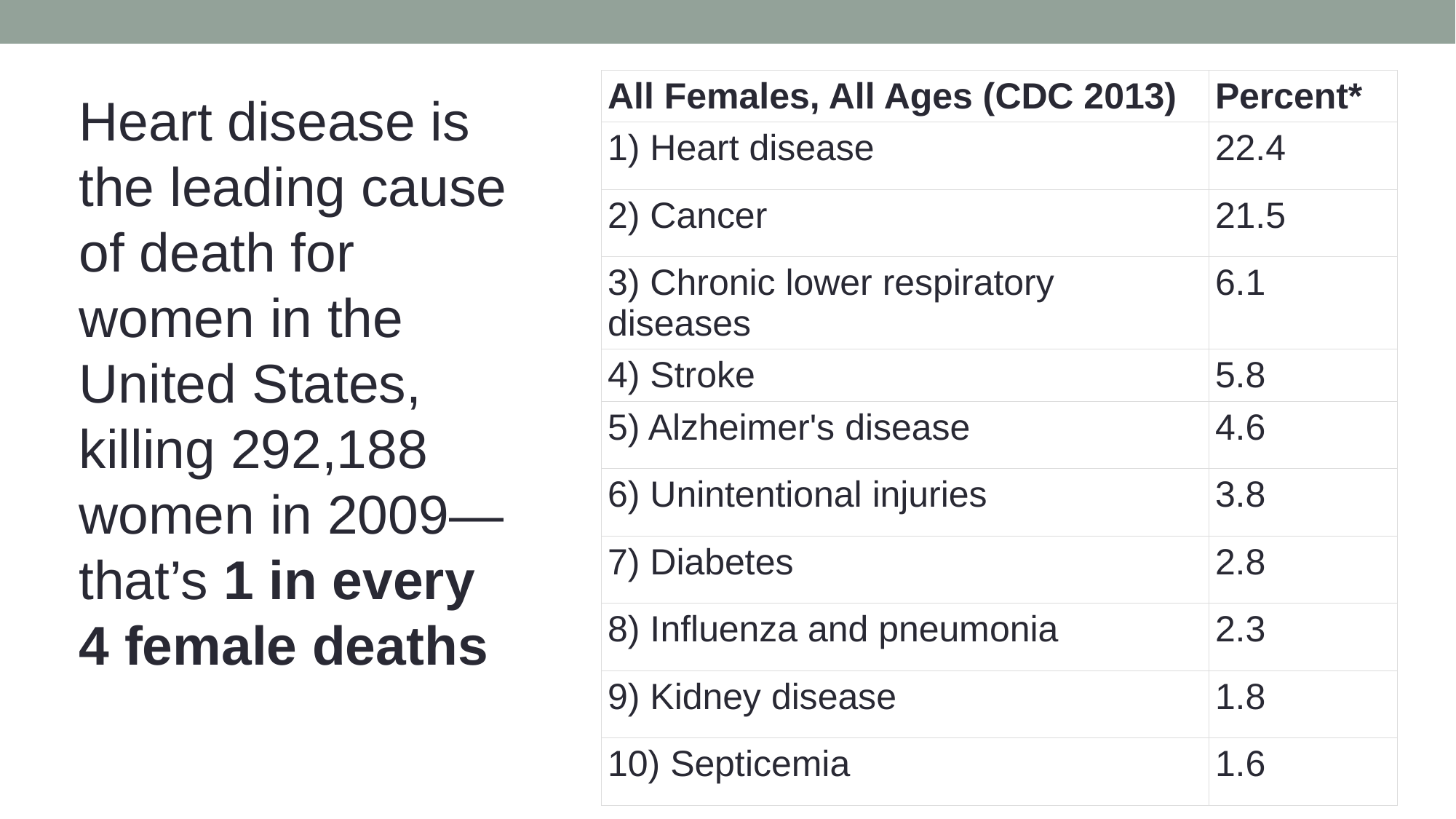

| All Females, All Ages (CDC 2013) | Percent\* |
| --- | --- |
| 1) Heart disease | 22.4 |
| 2) Cancer | 21.5 |
| 3) Chronic lower respiratory diseases | 6.1 |
| 4) Stroke | 5.8 |
| 5) Alzheimer's disease | 4.6 |
| 6) Unintentional injuries | 3.8 |
| 7) Diabetes | 2.8 |
| 8) Influenza and pneumonia | 2.3 |
| 9) Kidney disease | 1.8 |
| 10) Septicemia | 1.6 |
Heart disease is the leading cause of death for women in the United States, killing 292,188 women in 2009—that’s 1 in every 4 female deaths

## Slide 3
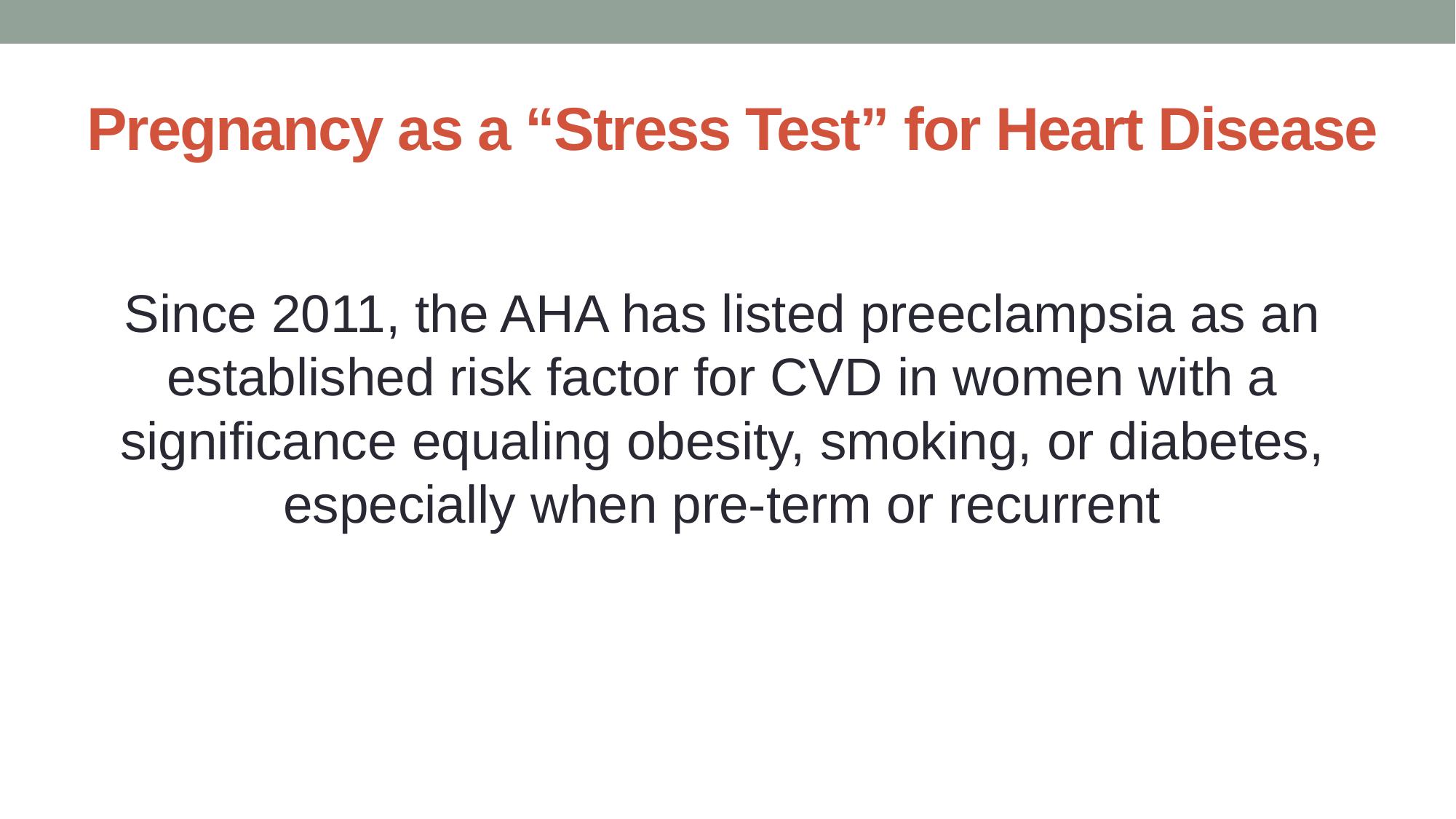

# Pregnancy as a “Stress Test” for Heart Disease
Since 2011, the AHA has listed preeclampsia as an established risk factor for CVD in women with a significance equaling obesity, smoking, or diabetes, especially when pre-term or recurrent

## Slide 4
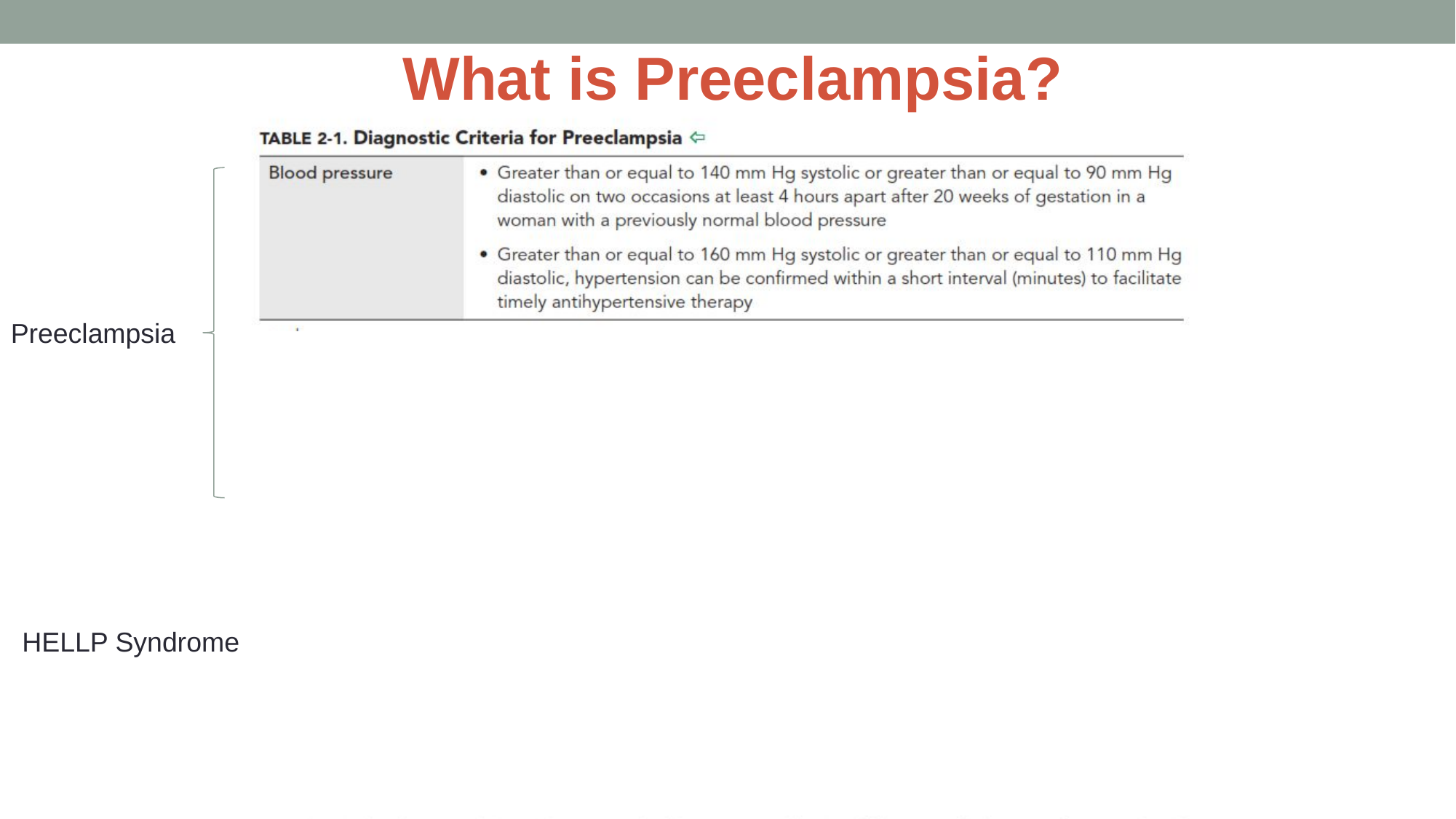

What is Preeclampsia?
Preeclampsia
HELLP Syndrome

## Slide 5
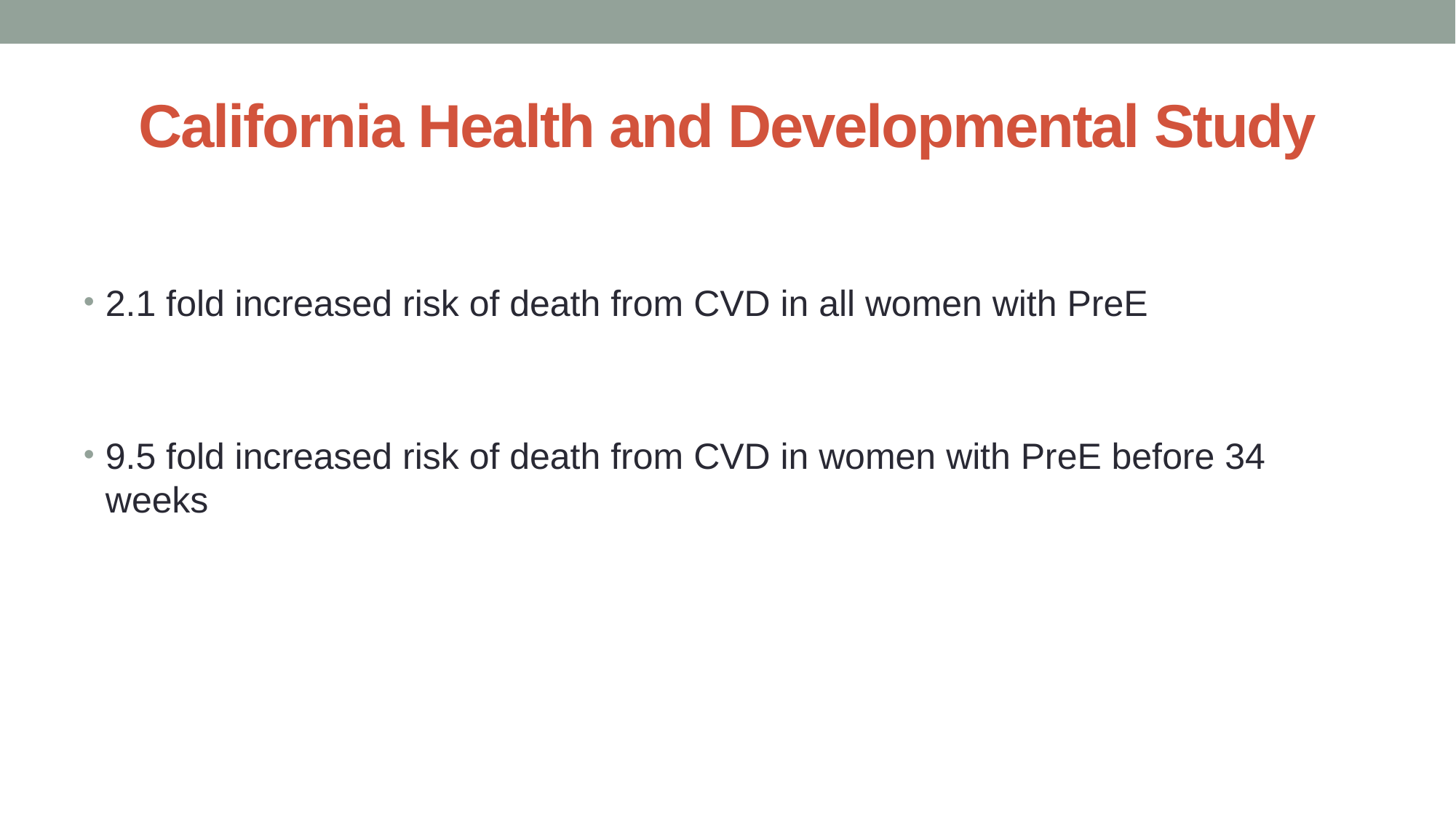

# California Health and Developmental Study
2.1 fold increased risk of death from CVD in all women with PreE
9.5 fold increased risk of death from CVD in women with PreE before 34 weeks

## Slide 6
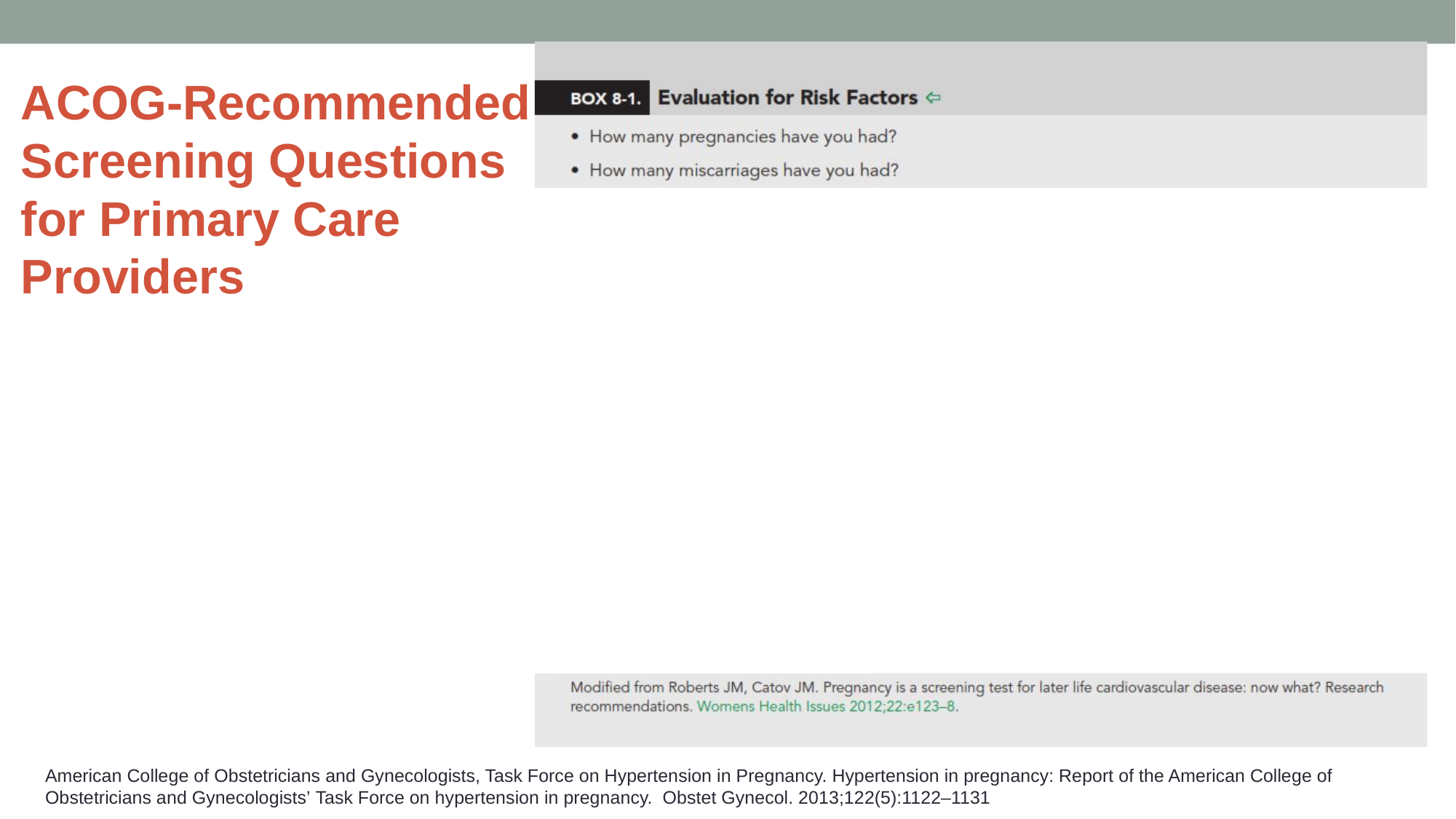

ACOG-Recommended Screening Questions for Primary Care Providers
American College of Obstetricians and Gynecologists, Task Force on Hypertension in Pregnancy. Hypertension in pregnancy: Report of the American College of Obstetricians and Gynecologists’ Task Force on hypertension in pregnancy.  Obstet Gynecol. 2013;122(5):1122–1131

## Slide 7
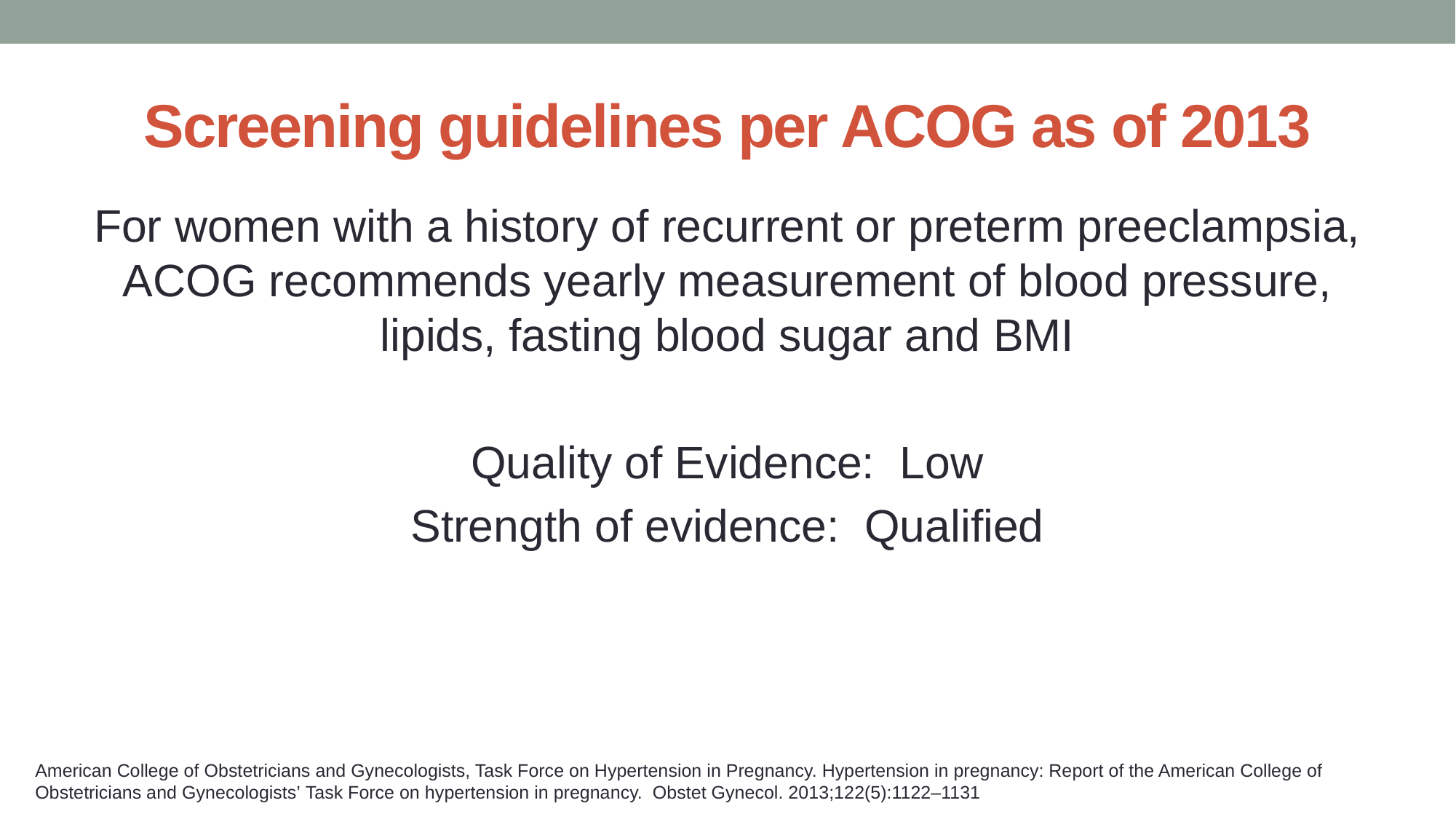

# Screening guidelines per ACOG as of 2013
For women with a history of recurrent or preterm preeclampsia, ACOG recommends yearly measurement of blood pressure, lipids, fasting blood sugar and BMI
Quality of Evidence: Low
Strength of evidence: Qualified
American College of Obstetricians and Gynecologists, Task Force on Hypertension in Pregnancy. Hypertension in pregnancy: Report of the American College of Obstetricians and Gynecologists’ Task Force on hypertension in pregnancy.  Obstet Gynecol. 2013;122(5):1122–1131

## Slide 8
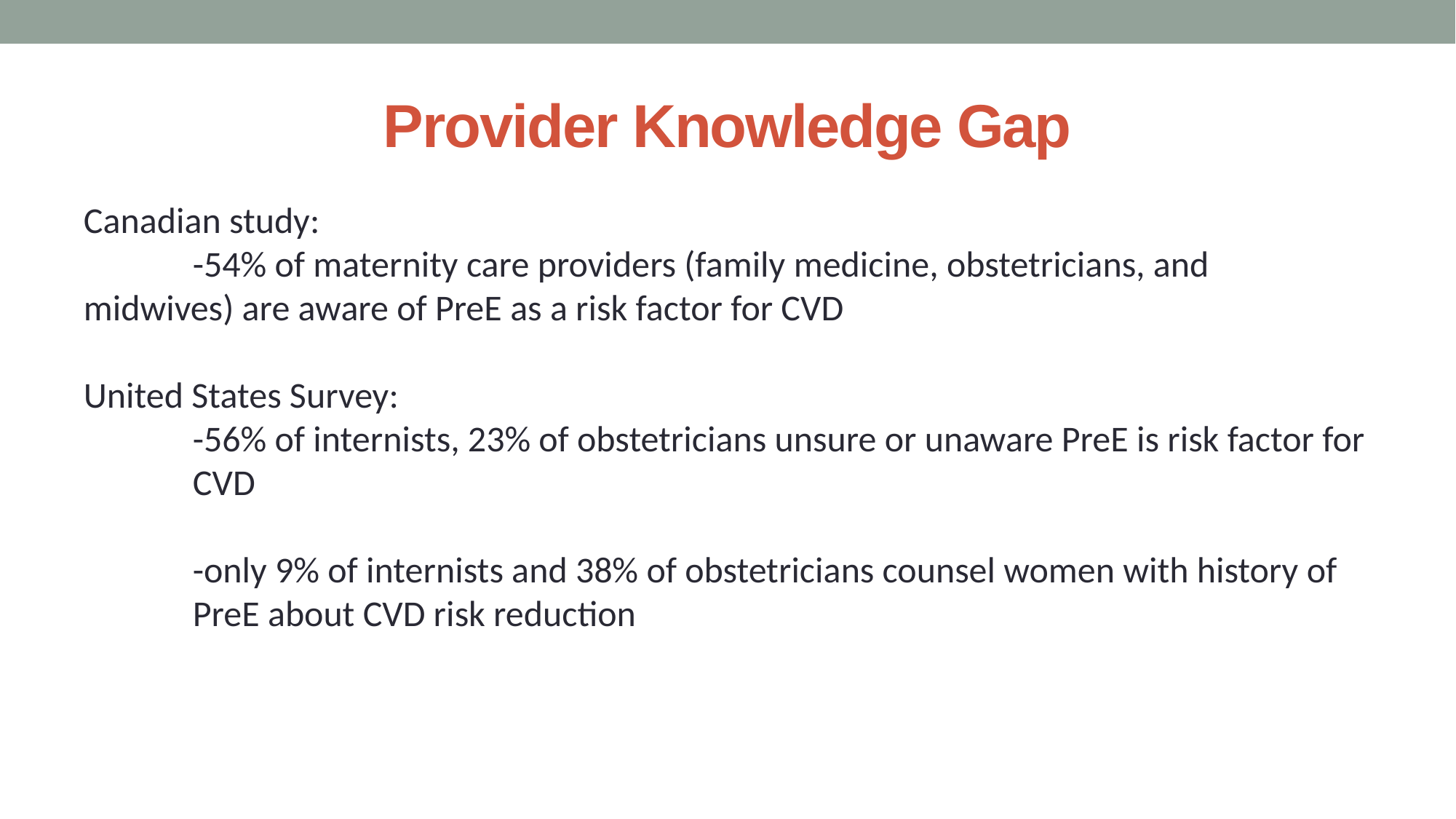

# Provider Knowledge Gap
Canadian study:
	-54% of maternity care providers (family medicine, obstetricians, and 	midwives) are aware of PreE as a risk factor for CVD
United States Survey:
	-56% of internists, 23% of obstetricians unsure or unaware PreE is risk factor for 	CVD
	-only 9% of internists and 38% of obstetricians counsel women with history of 	PreE about CVD risk reduction

## Slide 9
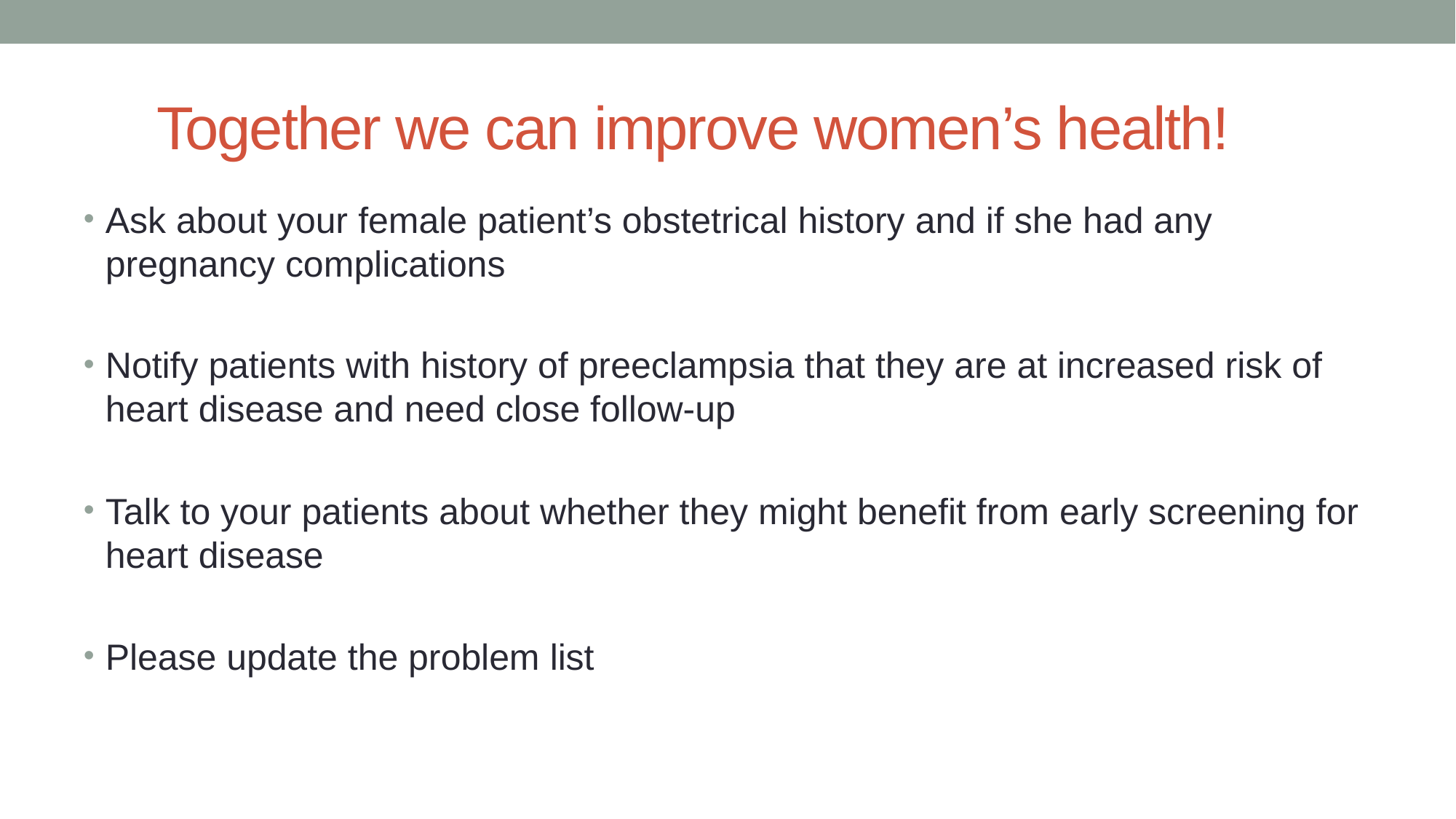

# Together we can improve women’s health!
Ask about your female patient’s obstetrical history and if she had any pregnancy complications
Notify patients with history of preeclampsia that they are at increased risk of heart disease and need close follow-up
Talk to your patients about whether they might benefit from early screening for heart disease
Please update the problem list

## Slide 10
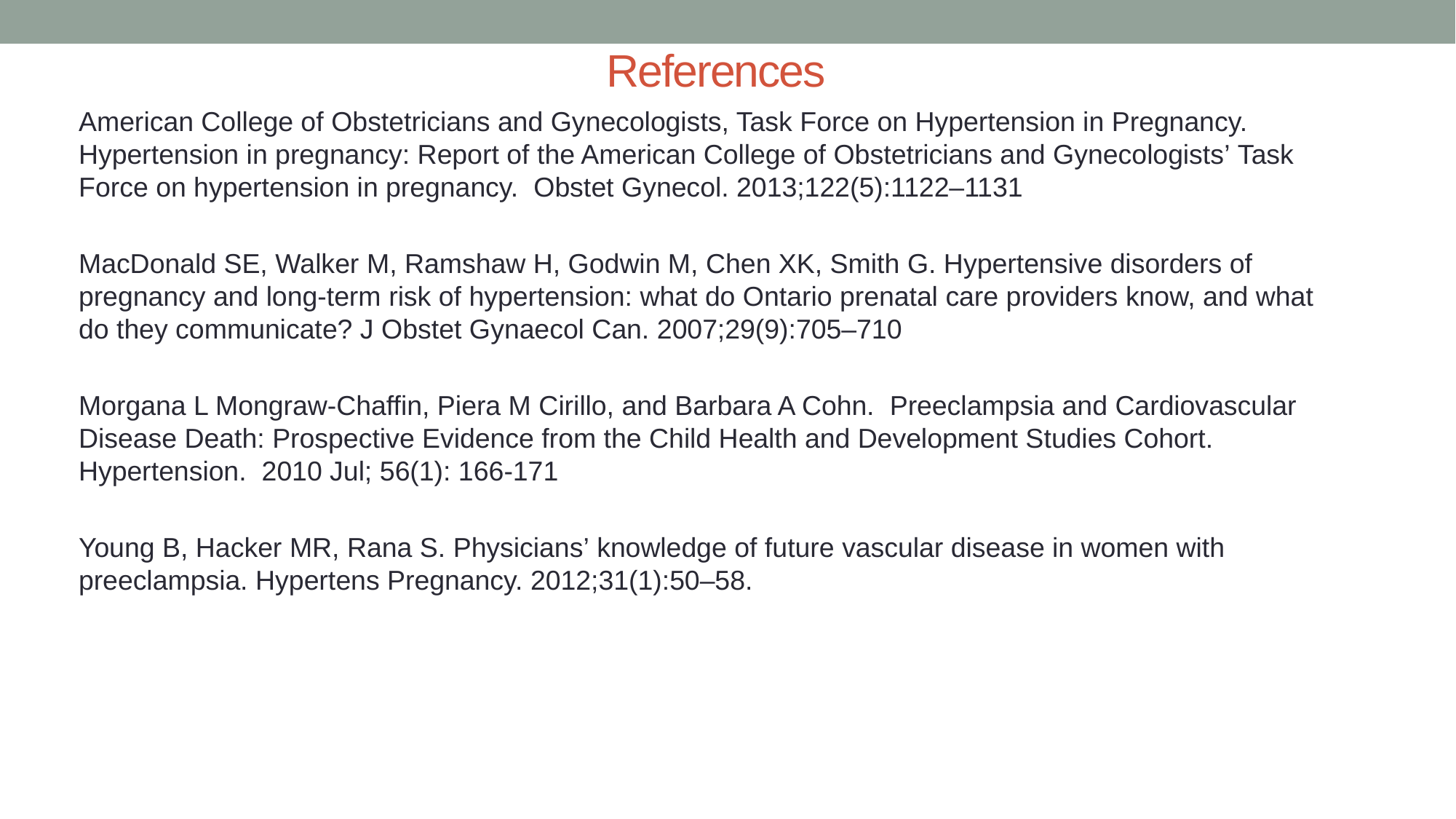

# References
American College of Obstetricians and Gynecologists, Task Force on Hypertension in Pregnancy. Hypertension in pregnancy: Report of the American College of Obstetricians and Gynecologists’ Task Force on hypertension in pregnancy.  Obstet Gynecol. 2013;122(5):1122–1131
MacDonald SE, Walker M, Ramshaw H, Godwin M, Chen XK, Smith G. Hypertensive disorders of pregnancy and long-term risk of hypertension: what do Ontario prenatal care providers know, and what do they communicate? J Obstet Gynaecol Can. 2007;29(9):705–710
Morgana L Mongraw-Chaffin, Piera M Cirillo, and Barbara A Cohn.  Preeclampsia and Cardiovascular Disease Death: Prospective Evidence from the Child Health and Development Studies Cohort. Hypertension.  2010 Jul; 56(1): 166-171
Young B, Hacker MR, Rana S. Physicians’ knowledge of future vascular disease in women with preeclampsia. Hypertens Pregnancy. 2012;31(1):50–58.
