# Supplementary material for: Missed opportunities to prevent cardiovascular disease in women with prior preeclampsia
Source: BMC Womens Health. 2020 Oct 1;20:217. doi: 10.1186/s12905-020-01074-7 (PMC7528479; doi:10.1186/s12905-020-01074-7)
Supplement: Supplementary file 3 — Additional file 3. Copy of email sent to internal medicine providers with PowerPoint slides attached. [file 12905_2020_1074_MOESM3_ESM.docx]

**Provider Email Including Educational Powerpoint Presentation**

*To be sent out to all internal medicine physicians at approved sites. This email will include the educational powerpoint presentation as an attachment, and will have a read receipt linked to each email. This read receipt serves only to confirm that providers have received and reviewed the material and track provider participation as a whole, but will not be used to track individual provider participation in order to preserve provider anonymity.*

Dear provider –

You’ve received this email because you are an internal medicine physician employed at Froedtert Hospital or an affiliated clinic. Through the collaborative efforts of Obstetrics and Gynecology, Internal Medicine, and Cardiology, we are conducting a study on Obstetric History Complications and invite you to participate. Participation in this study involves the following: a review of the brief presentation attached, and a read response to this email so that we can acknowledge the dissemination of the presentation to all internal medicine providers at Froedtert Hospital and affiliated clinics. The purpose of this study is to educate providers about the significance of certain obstetric histories and how these may be risk factors for later disease.

Recent data identifies a strong connection between hypertension in pregnancy and early onset cardiovascular disease, however there is no consensus on how to screen women with preeclampsia. The American Heart Association recognizes that preeclampsia is an independent risk factor for CVD, and ACOG recommends yearly HTN, lipid, BMP, and glucose screening. Unfortunately, our data demonstrates that in primary care we are not asking these patients about a prior history of preeclampsia, gestational hypertension, or gestational diabetes.

A short presentation is attached to this email. Please review this at your convenience. It provides important information on new recommended practices for screening the well woman for an increased risk of cardiovascular disease based on her pregnancy history. A read receipt has also been linked to this email. The read receipt will not be used to identify individual provider participation, only as a measure for tracking the overall percentage of providers who have received and reviewed the short attached slideshow.

Your participation is voluntary and anonymous, and will not impact your employment status. You will not be identified in any presentations or publications following your participation. If you have questions about your rights as a research participant or want to report any problems or complaints, you can call the Medical College of Wisconsin/Froedtert Hospital Research Subject Advocate at (414) 456-8844.

Thank you in advance for your assistance with this important project.

| Cresta W. Jones, M.D., FACOG  Assistant Professor, Maternal–Fetal Medicine  Director of Education and Professional Development  Department of Obstetrics and Gynecology  Medical College of Wisconsin  Office: 414-805-9019  [cjones@mcw.edu](mailto:cjones@mcw.edu) | Jennifer C. Mackinnon, M.D., MM Associate Professor  Associate Director, Ambulatory Quality Department of General Internal Medicine Medical College of Wisconsin  Office:  414-805-0819  [jmackinnon@mcw.edu](mailto:jmackinnon@mcw.edu) | Jacquelyn Kulinski, M.D.  Assistant Professor  Co-Director of Preventative Cardiology  Department of Medicine  Medical College of Wisconsin  Office: 414-955-6777  [jakulinski@mcw.edu](mailto:jakulinski@mcw.edu) |
| --- | --- | --- |
