# Supplementary material for: Missed opportunities to prevent cardiovascular disease in women with prior preeclampsia
Source: BMC Womens Health. 2020 Oct 1;20:217. doi: 10.1186/s12905-020-01074-7 (PMC7528479; doi:10.1186/s12905-020-01074-7)
Supplement: Supplementary file 3 [file 12905_2020_1074_MOESM1_ESM.docx]

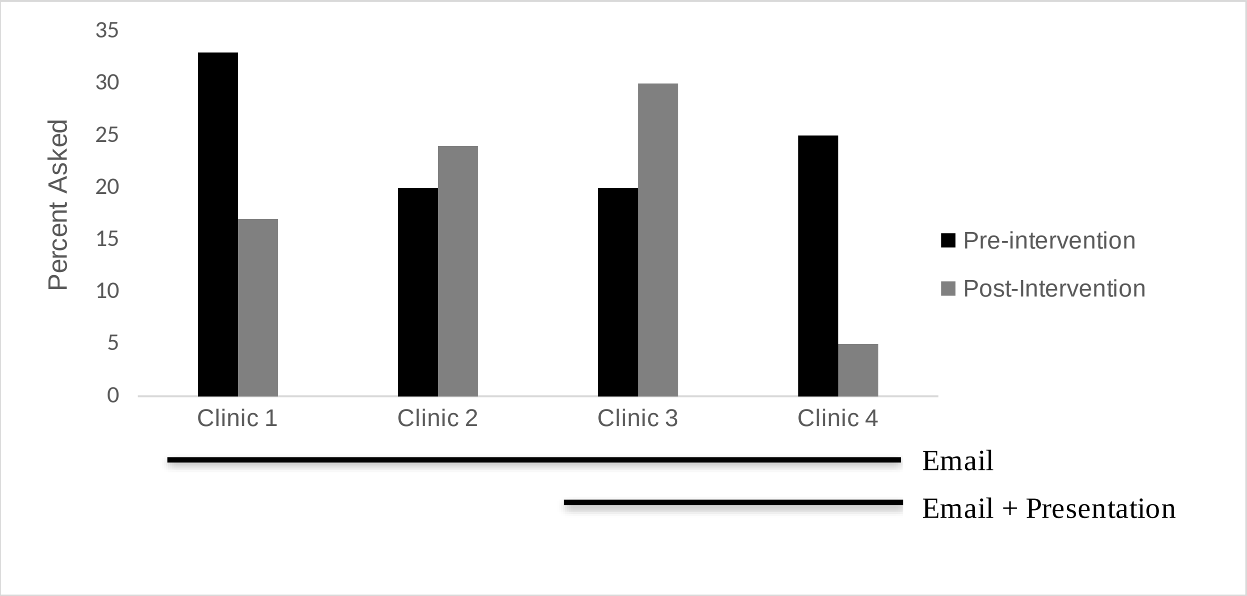
Percent Asked
35
30
25
20
15
10
5
0
Clinic 1
Clinic 2
Clinic 3
Clinic 4
Pre-intervention
Post-Intervention
Email
Email + Presentation
